# “As safe as possible”: a qualitative study of opioid withdrawal and risk behavior among people who use illegal opioids

**DOI:** 10.1186/s12954-023-00893-9

**Published:** 2023-10-27

**Authors:** David Frank, Luther Elliott, Charles M. Cleland, Suzan M. Walters, Paul J. Joudrey, Danielle M. Russell, Beth E. Meyerson, Alex S. Bennett

**Affiliations:** 1 Department of Social and Behavioral Sciences, School of Global Public Health, New York University, New York, NY 10003 USA; 2 Center for Drug Use and HIV/HCV Research, School of Global Public Health, New York University, New York, NY 10003 USA; 3 Center for Anti-Racism, Social Justice, and Public Health, 708 Broadway, 9th floor, New York, NY 10003 USA; 4 Department of Population Health, Grossman School of Medicine, New York University, New York, NY 10003 USA; 5 Department of Epidemiology, School of Global Public Health, New York University, New York, NY 10003 USA; 6 Division of General Internal Medicine, School of Medicine, University of Pittsburgh, Pittsburgh, PA 15213 USA; 7 Department of Family and Community Medicine, University of Arizona, Tucson, AZ 85711 USA; 8 Harm Reduction Research Lab, Department of Family and Community Medicine, College of Medicine, University of Arizona, Tucson, AZ 85711 USA; 9 Woodside, NY 11377 USA

**Keywords:** Opioid use, Withdrawal, Risk, Fentanyl, Disease model, Medicalization, Addiction, Harm reduction

## Abstract

**Background:**

Opioid withdrawal is a regular occurrence among many people who use illicit opioids (PWUIO) that has also been shown to increase their willingness to engage in risk-involved behavior. The proliferation of fentanyl in the illicit opioid market may have amplified this relationship, potentially putting PWUIO at greater risk of negative health outcomes. Understanding the relationship between withdrawal and risk-involved behavior may also have important implications for the ways that problematic drug use is conceptualized, particularly in disease models of addiction, which position risk behavior as evidence of pathology that helps to justify ontological distinctions between addicts and non-addicts. Examining withdrawal, and its role in PWUIO’s willingness to engage in risk, may aid in the development of alternative theories of risk involvement and create discursive spaces for de-medicalizing and de-othering people who use illegal drugs.

**Methods:**

This article is based on 32 semi-structured interviews with PWUIO in the New York City area who also reported recent withdrawal experience. Interviews were conducted remotely between April and August 2022 and recorded for later transcription. Data were then coded and analyzed based on a combination of inductive and deductive coding strategies and informed by the literature.

**Results:**

Participants described a strong relationship between withdrawal and their willingness to engage in risk-involved behavior that was exacerbated by the proliferation of fentanyl. Yet, their descriptions did not align with narratives of risk as a product of bad decisions made by individuals. Rather, data demonstrated the substantial role of social and structural context, particularly drug policies like prohibition and criminalization, in the kinds of risks that PWUIO faced and their ability to respond to them.

**Conclusions:**

Withdrawal should be taken more seriously both from an ethical perspective and as an important catalyst of risk behavior. However, theories that position activities taken to avoid withdrawal as irrational and as evidence of pathology are poorly aligned with the complexity of PWUIO’s actual lives. We recommend the use of less deterministic and less medicalized theories of risk that better account for differences between how people view the world, and for the role of socio-structural forces in the production of risk.

## Background

Opioid withdrawal is a regular occurrence for many people who use illicit opioids (PWUIO) involving acute physical and psychological pain that can sometimes be fatal [1–4]. A recent study shows that 85% of people who inject drugs reported experiencing withdrawal symptoms during the last 6 months and 35% experienced them weekly [1]. Withdrawal is also associated with overdose and receptive syringe sharing and is a barrier to safe injection and drug use practices [2, 5–7]. Despite this, there has been little investigation of withdrawal’s role in the lives of PWUIO and its relationship to risk, and almost none from the perspective of PWUIO. As Elizabeth Grey, a person with lived experience of heroin use, wrote in a recent Washington Post article, “The great, gaping hole of the response to the opioid epidemic is that withdrawal is the most important aspect, and it’s barely given lip service.”[8].

PWUIO have long asserted that withdrawal has been regularly misunderstood and devalued both by addiction professionals and within the public discourse [8–10]. They argue that comparisons to the flu as well as professional metrics such as the Clinical Opioid Withdrawal Scale (COWS) fail to capture the affective experience of withdrawal. As Frank wrote in a recent article calling for greater reliance on the views of people with lived experience of drug use, such measures “fail to capture the feeling of one’s Central Nervous System having lost the ability to regulate how we experience the world, its capacity to make our existence feel more-or-less comfortable. As a result, such metrics and comparisons consistently mischaracterize and underestimate withdrawal’s capacity for producing dysphoria and pain.” [9].

Attention to withdrawal’s role as a catalyst for risk may be particularly important now because of the proliferation of fentanyl and related rise in overdose rates [11–13]. Fentanyl, a highly effective mu-opioid receptor agonist, estimated to be 50–100 times more potent than morphine, began to appear in the illicit opioid supply in approximately 2008–2013 and has since proliferated rapidly [14–16]. The Drug Enforcement Agency’s National Forensic Laboratory Information Service reported an over 1000% increase in fentanyl reported in drug seizures from 2014 to 2017 [14]. Because of its high potency in relation to heroin [17, 18], Fentanyl use leads to greater tolerance and thus, the need to use opioids more often and in greater amounts to avoid withdrawal [19, 20]. As such, the withdrawal produced from regular fentanyl use may have a greater impact on PWUIO’s willingness to engage in risk-involved behavior.

Understanding the relationship between withdrawal and risk-involved behavior is also important because of how such behavior has been conceptualized in theories of addiction-as-disease like the brain disease model. Disease-based models, particularly in the USA, have typically framed the propensity of “addicts” to engage in risky activities as evidence of pathology, reflecting a lack of autonomy and rationality that is seen as the essence of addiction [21–25]. For example, John Foxe, chair of the Department of Neuroscience at the University of Rochester Medical Center states “The vast majority of people, when faced with something they want, will assess how achievable the goal is and adjust their actions and expectations in order to maximize their potential to achieve it. However, it appears that the integrity of this system of assessment and self-regulation is impaired in substance abusers and this may contribute to the risk-taking behaviors and poor decision-making commonly associated with this population.” [25].

The same kinds of claims also appear frequently in lay discussions of addiction where metaphors of possession and hijacked brains are regularly deployed to illustrate putative addicts’ lack of rationality and autonomy [23, 24, 26, 27]. For example, in this 2018 *New York Times* story titled, “Heroin Addiction Explained: How Opioids Hijack the Brain” the writers describe the process as follows: “THE BRAIN SCREAMS for more. Scoring the next fix feels like a race against the clock of withdrawal. You may feel like only a fix can save you. It makes no sense, but this compulsion takes over all logic, judgment and self-interest. You may do things you never thought you could.” [28].

As such, behavior seen as risk-involved has been used both as evidence of addiction and to justify ontological distinctions between putative addicts and everyone else [24, 25, 28, 29]. Yet, this view of risk has also been challenged by scholars who argue it falsely assumes a universal metric for assessing the rationality of peoples’ decisions that ignores cultural and individual differences in how we perceive risk [30]. For example, proponents of the Rational or Liberal model of addiction have argued that people who use drugs, like most people, make decisions according to their own values and worldview [21, 22, 31]. Sociocultural perspectives have also shown that the way that withdrawal is experienced is closely linked to how it, and other, related concepts like “addict,” “clean,” “recovery” are social constructed [7, 32, 33].

The “risk environment” framework, developed by Tim Rhodes, is a useful means for problematizing the idea that risk emerges solely from bad decisions made by individuals. Rather than focusing strictly on individuals to understand risk, the risk environment framework posits risk as produced through an interaction between individuals and their socio-structural context. [34–36]. Rhodes defines the risk environment as “the space—whether social or physical—in which a variety of factors interact to increase the chances of drug-related harm” and describes the relationship between harm and its environmental context as one of “contingent causation” [35]. The risk environment framework is also a response to public health’s tendency to focus on risk as a product of individuated factors and at the expense of social and structural context [35–37]. Rhodes points out that such models, which are usually based on theories of “rational decision-making” and “reasoned action,” are poorly suited to understanding individual behavior within the larger sociocultural and structural environments in which decisions about health and risk are made [35]. Moreover, by focusing primarily on the individual, public health narratives tend toward victim-blaming and can easily be used to pathologize people who use drugs. In contrast, the risk environment model starts from the premise that behavior occurs in a social world where risks are both relative and related to the social, cultural, economic, legal, policy, and political environments that they occur within. As such, interventions and policy recommendations that emerge from a risk environment analysis are less likely to be used to blame and discipline people who use drugs and are better equipped to address “non-drug and non-health specific factors.” [35, 38].

Opioid-related morbidity and mortality remains one of the most pressing public health crises of the past 100 years, and overdose rates continue to rise among PWUIO [39–41]. Examining withdrawal, and its role in PWUIO’s willingness to engage in risk, could inform clinical and harm reduction strategies to reduce overdose risk behaviors and other harms among people who use drugs. A better understanding of the role of withdrawal in PWUIO’s lives may also aid in the creation of alternative theories of risk involvement and create discursive spaces for de-medicalizing and de-othering people who use illegal drugs. As such, this article uses the risk environment framework to better understand withdrawal in the lives of people who use illegal opioids; understand the relationship between withdrawal and risk; and inform evolving constructions of withdrawal.

## Methods

### Sample and recruitment

This article is based on data collected from 32 semi-structured interviews with PWUIO in the New York City area who also report having experienced opioid withdrawal at least once during the past 30 days. Our sample was 50% male (*n* = 16), 46% female (*n* = 15), and 4% who did not disclose their gender (*n* = 1). Racially, our sample was 40% white (*n* = 13), 25% Black (*n* = 8), 28% Latinx (*n* = 9), and 6% who did not disclose their race (*n* = 2). The mean age of our sample was 40.8.

Participants were recruited using a purposive strategy from an ongoing longitudinal study that examines overdose risk management in the era of Naloxone (*Overdose Risk Management and Compensation in the Era of Naloxone* (PIs: Bennett and Elliott)). Participants in that study had the opportunity to give consent to be contacted for future research studies, and those who did so, who also met the study criteria, were contacted by email and/or text and asked if they were interested in participating in an additional interview that would seek information on themes that were closely related to those in the original study. We also sought to include participants who represent a diversity of ages, racial and ethnic categories, and a variety of locations.

### Data collection

Interviews lasted approximately 60 min and were conducted remotely using the host university’s HIPAA compliant Zoom teleconferencing platform. All interviews were audio recorded; video was also recorded when participants expressed comfort with including video. Interviews covered participants’ experiences in Medication for Opioid Use Disorder (MOUD) over time and their experiences with withdrawal both while in and not in MOUD; how, if at all, withdrawal impacted their experiences with drug use and risk behavior; and their experiences with overdose, naloxone, and use of OnPoint, the NYC overdose prevention center.

Participants were compensated $50 via their Clinical Trial Payer (CTPayer) incentive cards at the completion of each interview, and the audio was then transcribed by a professional transcription service. All participants provided informed consent and are referred to by pseudonyms. The study was approved by the New York University Langone Institutional Review Board.

Interviews were conducted using a situated approach based on Dr. Frank’s lived experience using illegal opioids and in MMT. Situated approaches are those that recognize the positionality of the researcher in regard to the object of study and strive to be transparent about those relationships rather than to eliminate bias [42]. Dr. Frank disclosed his own status as someone who has used illegal drugs and who is currently in MMT to participants. Although he has discussed many of the methodological issues associated with this choice in other articles (see, for example, Frank and Walters, [43]), in short, the authors believe that by disclosing Frank’s shared history with participants, he was able to develop a level of comfort and trust that facilitated more robust and honest conversations and a richness of data that would not have been possible otherwise. Community-driven research with people who use drugs has documented the mistrust that people who use drugs often feel toward public health and substance use researchers and similarly recognizes the importance of research conducted by and involving community insiders [44–47]. This approach has also been used successfully elsewhere [48–50].

### Analysis

Interviews were coded by Dr. Frank using Atlas.ti based on a combination of inductive and deductive coding strategies. Reliability was assessed at multiple points in the analysis based on discussions with co-investigators (Bennett, Elliott, and Cleland) and in-line with practices established by similar qualitative, exploratory projects [51, 52]. Analysis was guided by a thematic approach that aimed to organize data into meaningful categories based on the aims of the study, the tenets of the risk environment framework, and existing literature [53, 54]. The risk environment framework was chosen for this analysis in an iterative fashion by the authors during post-interview discussions based on its utility toward understanding study themes. However, our analysis does not identically mirror Rhodes typology which includes four specific environments—physical; social; economic; and policy—organized into macro- and micro-levels of influence, and which he states are intended as ideal types to be used as a heuristic rather than discrete categories with specific relationships. Instead, we based our analysis on Rhodes’ central argument that risks associated with drug use should be understood by situating individual behavior within its sociocultural context and used his typology as a guide.

## Results

### Withdrawal and risk in the era of fentanyl

Participants emphasized both the pain of withdrawal and its ubiquity in the lives of PWUIO. They described periods when they were not on medication for opioid use disorder (MOUD) as characterized by a near-constant effort to prevent and avoid withdrawal. Many reported regularly feeling sick upon waking up, even if they had used opioids the previous evening and described attempting to “stay well” as a constant challenge. For example, participants stated:

“I honestly feel like I've been sick every fucking day, every couple of hours, it's fucking hard.”

-Thomas (white, male, 31 years old)

“Let me say that, okay, today's Tuesday. For example, I'll use today. I'm back home by two or three and like by six, between 6 to 10 hours, I'll start getting withdrawals.”

-Gina (Latinx, female, 47 years old)

“In the past two months, every day I was waking up sick because I've been doing like three bags a day now.”

-Charlene (white, female, 30 years old)

Participants specifically emphasized how the proliferation of fentanyl has exacerbated their experience of withdrawal. They described the withdrawal symptoms from regular fentanyl use as “heightened” and “stronger” than those they experienced before fentanyl began to replace heroin in the illicit opioid market [in NYC] and reported that fentanyl habits required more frequent use to avoid withdrawal. Not surprisingly, this created a greater sense of anxiety and urgency to find relief. For example, participants reported:

“The withdrawal symptoms are different. They're heightened, and it's stronger. It's, I don't know, because when I used to get sick about heroin, it was almost like I could deal with it. It was vomiting and the chills and stuff but with this? Oh no.”

-Doreen (Latinx, female, 38 years old)

“It's getting worse [Since fentanyl become the dominant opioid]. I've noticed every time it [withdrawal] takes longer and I go through more added symptoms that I've never been through before. It's getting worse. Yeah and [with fentanyl] that's a different battle. I don't think that they ever were prepared to deal with that battle... I think they just think ‘oh he's just withdrawing’. It's serious man.”

-Isaiah (Black, male, 55 years old)

Participants’ responses also reflected a sense of frustration with how opioid withdrawal has been conceptualized both in general and particularly by addiction professionals. Many brought up the often-used comparison of withdrawal to the flu as evidence of the way that withdrawal experiences have been minimized and devalued in addiction discourse. As Andria stated:

“Oh my God, it's so much different from the flu. You are throwing up and nothing is coming out, and then I get this thing in the back of my throat where it's like dusty, where I can't even swallow. Oh my God, and then I feel like I'm really going to die. In seconds you are like ‘please, please just let me get through this’. I can laugh about it right now. Yeah, [Addiction doctors and people that research drug use] they don't understand. They think it's like the flu.”

-Andria (white, female, 30 years old)

Thus, responses demonstrate the ubiquity, pain, and dysphoria of withdrawal in PWUIOs’ lives and particularly since the proliferation of fentanyl which made withdrawal experiences both more common and more severe.

Participants also described withdrawal as an important catalyst for engaging in risk-involved behavior. In fact, many reported that whether they were in withdrawal often determined whether or not they would engage in particular activities. Participants reported a variety of risk-involved activities that they linked to withdrawal, including stealing from family and friends, committing violent crimes, and unwanted and/or particularly risk-involved forms of panhandling and sex work. For example, participants stated:

“When I was sick there were certain things that you wouldn't do, and then you were sick and you do it. When it came down to get out of pain, I made a lot of decisions that I wouldn't have made. That I wouldn't do if I wasn't dopesick.”

-Gustavo (Latinx, male, 40 years old)

“I've done things that I'm not proud of, you know? I mean, I would hustle over my family, you know, and I still feel guilty about that. [If I had not been in withdrawal] I would never do that to anybody. My family? never. They would help me out, you know? They would know and not say anything. Now I burned that bridge.”

-Andria (white, female, 30 years old)

Withdrawal was also seen as an important driver of less safe drug use practices, including sharing syringes, using larger amounts of opioids, and being less likely to use fentanyl test strips or other safer injection practices. Participants reported:

“I got hepatitis that way [sharing a syringe when I was in withdrawal]. And the fucking girl told me she had it too, and I said ‘okay’. Yeah, I was well aware, but we were in the middle of fucking nowhere and that was the only fucking syringe, and that was that.”

-Kelly (white, female, 26 years old)

“Yeah, I do [use the fentanyl test strips]. But I tell you, when you go through withdrawals, then not really.”

-Aliyah (Black, female, 46 years old)

“If you're sick man, you're just trying to get that shit in you. If I don't have any help, I can't even do a shot until I sniff a bag because my nerves are shot. I'm just so fucked up. Yeah, I'll use the same one [syringe] and not even worry about it. Instead of doing two bags, you do three.”

-Julia (female, race not identified, 36 years old)

Since participants knew that withdrawal made them more susceptible to risk, many reported efforts to avoid it by planning ahead, rationing their supply of drugs and money, and accomplishing necessary tasks before withdrawal symptoms became too debilitating. For example, participants stated:

“Well, like I'll do what I have to do, like to get money. I try to do it before I'm gonna be sick because the thought of being sick is like, it's like the worst feeling in the world to me. When I'm feeling sick that my energy level is like down, going out to make money is like ‘oh shit’ you know?”

-Shauna (white, female, 48 years old)

“If it didn't seem like she [my drug seller] was going to be able to do it tomorrow, I would go out and boost [steal] while I still wasn't sick, you know, because, like, I...I never...I was against boosting while you're sick because that's how you get arrested... you need it too badly; like, even if you're hot, even if they're on you, you still might try to push it, which is crazy.”

-Andrew (Black, male, 30 years old)

However, because withdrawal could only be put off for a relatively short amount of time, particularly when using fentanyl, this eventually proved impossible for most. PWUIO generally agreed that there was no way to consistently avoid withdrawal and the risks it engendered. As such, their comments reflected a particular worldview whereby exposure to risk was seen as an inherent, and unavoidable, part of being a PWUIO. Risks could be managed, and indeed they required constant management but could never be avoided entirely. For example, when asked about strategies for avoiding risk-involved drug use, one participant stoically responded, “I use, I take a chance”; another answered, “I try to be as safe as possible.”

### The role of drug policy in shaping risk decisions

Although participants often framed risk decisions individually (a not surprising outcome considering the dominance of individuated narratives of causality within health discourse), their responses aligned with Rhodes notion of contingent causation by demonstrating the importance of context in driving risk behavior. The role of drug policy, particularly prohibition/criminalization in creating dangerous conditions for PWUIO, and as a force that constrained their ability to respond to risk was particularly notable. This was demonstrated first through participants’ descriptions of fentanyl use. Most reported a clear preference for pre-fentanyl heroin and knew that currently available fentanyl/heroin increased the frequency and severity of their withdrawal experiences. Yet, because of market conditions associated with prohibition that PWUIO had no control over [55–57], their choices were limited to using fentanyl or nothing at all. Moreover, most stated that having developed a tolerance to the fentanyl/heroin, pre-fentanyl heroin would no longer be sufficient to ward off withdrawal. As Elana explained:

“Yes [you have to use more often], that's why I'm in withdrawal more because there's only two people that have the same stuff that has 90% fentanyl that we've been doing and we didn't even know. When they're not around we are fucked because the good dope, the good shit back in the day, does nothing to us, and we're still sick because these motherfuckers got us addicted to fentanyl without us knowing.”

-Thomas (white, male, 31 years old)

Responses also illustrate how the illegality of heroin/fentanyl restricted participants’ choices to a chaotic and unreliable illegal market that made withdrawal more difficult to avoid and thus impacted their substance use decisions. For example, Julia reported:

“Wondering when you're going to get the next bag, worried about the next bag and stuff. It gets testy out there and yes, I've done things that I'm not proud of just to actually secure that next bag. Just knowing that two minutes ago I wasn't feeling good and what I had to do to get this $10 and, oh my God, in a few hours he's not going to be around till six in the morning, and then what am I going to do at six in the morning? So now I got to go do something stupid.”

-Julia (female, race not identified, 36 years old)

Not surprisingly, participants reported constant stress and anxiety over the need to consistently obtain opioids and stay one step ahead of withdrawal, which was always only a few hours away. This meant that PWUIO had to engage in progressively more and more risk-involved activities to stave off or find relief from withdrawal, as described here by participants:

“It was chaotic right, 'cause I needed to get the money, you know, and get the money and then find the drugs. I dealt with the same guy, right? And he would run out and then I'd be all messed up because I had to find somebody else. And to find somebody else was a job because then they don't want to sell to you. They don't want to sell to you because they don't know you.”

-Andria (white, female, 30 years old)

“Yes, if I cannot get from my regular, and I know my regular is not going to be here anytime soon and it's going to get past the aches and the muscles. If I know I'm already close to that, yeah, I'm going to get whatever I can and just pray to God that it's going to at least take away the sickness. And it's happened plenty of times, [that I got] street garbage, it did nothing and then I felt even 10 times worse and I had to run right back out to the corner and stand at the deli or supermarket to make another $20 and then wait for my regular to come?”

-Julia (female, race not identified, 36 years old)

One participant who sold heroin/fentanyl to avoid withdrawal described how the transition to primarily fentanyl drove him to participate in more risk-involved activities. Isaiah reported:

“It just makes me feel like a hamster when I'm trying to sell dope [fentanyl] because I can never keep enough to sell because I'm always doing it. So I found that stealing, it works out for me more even though it's more of a risk.”

-Isaiah (Black, male, 55 years old)

Participants’ responses also demonstrate the ways that prohibition/criminalization creates an environment where PWUIO have to manage multiple risks at once, and that the best response to one risk did not always align with that of another. For example, the need to avoid law enforcement often took precedence over safe injection concerns. This was particularly true for PWUIO who were homeless or who otherwise lacked a safe place to use drugs; however, most participants described the need to avoid law enforcement as an important part of their risk calculations. The following comments reflect these difficulties:

“Listen, when I'm going out dope sick and going to the those spots to get it, once I have that bag in my hand, I'm opening it up right there, and so, I'm doing it right there. One, it gives me the chance to sniff it and if I get caught, I don't have nothing in my hands.”

-Gina (Latinx, female, 47 years old)

“I mean I look for somewhere that's like it's discreet as possible. Which is it's hard in New York for sure. There's places where there's always people using and that seems like a slightly better idea than just to be by myself. Because then if I guess if police do roll up or something, I'm not the only one there like kind of sneak away or something. I usually just throw a blanket or a shirt over my head..”

-Laura (white, female, 23 years old)

“I'm homeless and I'm outside and usually being outside and using drugs, you will go to jail.”

-Elizabeth (white, female, 26 years old)

Thus, risk decisions were not perceived by participants as binary choices between whether or not to take a particular chance—such as using drugs in public—but as a constant process of evaluating and negotiating with multiple kinds of risk, with the overall aim of day-to-day survival. Since participants were well aware of the link between withdrawal and risk behavior, obtaining drugs to avoid withdrawal was itself seen as a strategy for avoiding risk.

### The role of participants’ economic situations in shaping risk decisions

Responses also evinced a strong relationship between an individuals’ available economic resources and their need to participate in risk-involved activities. This was most evident among people who were homeless or housing insecure. For example, Sandra reported:

“I was homeless, I was using to endure the homelessness. I was boosting [stealing] to get money, to get drugs and just everything was just very chaotic. We were living just day by day, one second at a time and everything was just very insane. Everything sucked and that fear of going to sleep every night with that one little bit of a bag left being like ‘shit, what I'm I going to do tomorrow’?”

-Sandra (white, female, 29 years old)

Participants also described an evolving process whereby the longer they had been using, the less available resources they had to moderate the risks of illegal drug use. For example, Doreen, a 38-year-old woman who recently began a MMT program, stated:

“Yes [I take more risks recently], because I have to do things, risky things in order to get it [heroin] now because it's like I'm coming to the end of my rope, so that's when you have to go for help, something's got to change. I went from having money to just one day not having it. That's a big adjustment, I was going from my two bundles a day, so [now] I can't even get two bags. That's a problem. Or just things like that, unprotected sex, things I don't want to have to do. I want to be left alone, a loner, like one of those. It's hard because then I got to put myself in groups of people that don't mean me any good. I just got caught yesterday stealing from the store, freaking things like stealing.”

-Doreen (Latinx, female, 38 years old)

In contrast, participants who had economic resources were much better equipped to avoid or address withdrawal without having to take as many risks. For example, Lawrence, one of the few participants who described himself as financially stable while using heroin reported a relatively risk-free life. He stated:

“I wasn't [taking risks] actually, cause I was what is considered a functioning addict. I worked; I had money. I very rarely had to do that.”

Lawrence (White, male, 29 years old)

However, most participants were financially insecure, partly because of managing the difficulties of illegal opioid use, and many emphasized the need to maintain their job or school attendance. As such, withdrawal, which could lead to missed work, school, etc., and could alert employers or coworkers to their drug use, had to be avoided as much as possible. For example, Andrew reported:

“I got too high into the debt with my checks and stuff like that. So, I would have to go [to work]. Sometimes, on the week that I would get paid, you know, I would have to go, like, the last two days before my check without it [having used opioids]. That shit would suck. And, like, yo, I'm there and then people are asking me like, ‘yo, why are you yawning so much’ Like, ‘why are your eyes watery?’”

-Andrew (Black, male, 30 years old)

Participants also described the need to avoid being seen in withdrawal by family members, with whom relations were often strained, and who often believed that participants were not currently using drugs. Family members were also often providing financial support and/or living space to participants, which could be jeopardized by appearing to be in withdrawal. As Gina explained:

“I tried to stay home sick and saying okay, ‘I'll stay home and [be] dope sick, [but] I cannot sit in my house, do anything, you know. It's one of the problems I had, experience with my family. They don't know anything about the heroin, you know, and not having no money and being dope sick, it's very chaotic for me.”

-Gina (Latinx, female, 47 years old)

The following interview portion illustrates how the difficulties involved with trying to manage multiple forms of risk can lead to negative outcomes such as overdose. As Mike explained:

“I didn't want to go into the office [while in withdrawal]. I was working for a magazine. So I made up this whole thing, I want to stop and get breakfast before work and I was [actually] meeting my guy [dealer] at McDonald's, and yes, it was bad. The guy had told me it was very strong and I insisted on going into the bathroom in McDonald's and I fell out [overdosed] in there. I don't remember the situation because I fell and hit my head. Yes, it was just, it was bad because I had my boss waiting outside.”

-Mike (white, male, 32 years old)

Thus, participants’ need to engage with risk-involved activity was contingent on their economic status and ability access economic resources. For some, avoiding withdrawal, even if it involved having to take risks by obtaining and using drugs, was often perceived as safer than the alternative, which could lead to loss of housing and income at a time when they already lacked stability and resources.

### The role of social context, setting, and norms in shaping risk decisions

Participants also emphasized the importance of social context in how they responded to withdrawal. Individuals who had greater social capital or access to social networks of people who use and/or sold drugs were sometimes able to avoid withdrawal by obtaining drugs, either from friends or a drug seller who would provide them with drugs “on the front.” Those with strong social networks were also able to ask friends about which drug-sellers to buy from if their regular source was unavailable. As such, they were less vulnerable to risks associated with having to obtain money illegally and/or buying from unknown sources. For example, the following participants stated:

“Let's say, oh, they say it takes 3-4 days before it [withdrawal] starts going away, but I'm like, ‘sheesh, four days’ But you know, I had friends that helped me out.”

-Mateo (Latinx, male, 58 years old)

“I used to call my friends, I have a lot of friends that sell drugs. [I would say] ‘Listen man, you know, I'm fucked up right now’, you know, or ‘I'm waiting on my unemployment’ or ‘my group gets paid Friday’, you know. ‘I'll give you extra 10 dollars man’.”

-Gina (Latinx, female, 47 years old)

Similarly, participants who had access to friends and family who would loan them money were also better equipped to avoid or address withdrawal.

“I was good at avoiding it, you know what I mean? I was good at avoiding it. I would like plan ahead, so, like if my money was going to be running out today, I'm already, like, you know, calling all my friends in PA or DC or my grandmother to get something at least set up. I’m a good, you know, telling stories, you know, twisting the truth.”

-Andrew (Black, male, 30 years old)

Location and setting also impacted the amount risk associated with particular activities. People who did not have a safe and private place to use were less able to take their time and adhere to safe injection protocols when preparing and using their drugs. For example, although Erin, a 26-year-old, gender-unidentified participant reported injecting more quickly and in greater amounts when in withdrawal, they emphasized that adhering to safer practices became nearly impossible in public places:

“I think what a big part of it too is just like not having a safe place to use all the time. You know you're like in a bathroom, somebody's pounding on the door and you're like ‘shit, oh shit’ and you just like bite off a bigger chunk or do a little more than [you normally would] and they're like, ‘oh whatever it'll be fine, no big deal’. And then next thing you know it's not fine.”

-Erin (white, gender not identified, 26 years old)

Similarly, responses emphasized how the need to conform to social conventions incentivized people in withdrawal to use drugs in risky ways. For example, participants described how withdrawal symptoms could include loss of bowel control, vomiting, uncontrollable sneezing, crying, and other publicly unacceptable behavior. Participants, many of whom already felt stigmatized and who had experiences multiple forms of trauma, were particularly vulnerable to fears of public embarrassment which could also draw the attention of law enforcement. As Kelly explains in the following interview segment:

“I was sick once and the bus was going to come, it was going to be the last bus. And I had like 20 minutes and it was a dead area, so I was like, I'm going to sit here, and then it was a very crowded bus and I sat right there on the bus and I used on the bus I couldn't wait. I couldn't wait 25 minutes to my fucking stop. Or even just to the next stop. I was like, fuck it I'll just go to the next stop, but the next stop was too far away from me too. I knew there were people staring at me and I didn't fucking care. Like, I didn't fucking care and I could have just waited 20 minutes for it, but, no I can't. Until we get any better, you can't wait 40 minutes…. I also have like a couple of, I don't know. I get this, like this really weird symptoms on top of the regular withdrawal symptoms and it's really unbearable. It's a really strange thing and it's so like that's the number one thing that really fucked with me. But my whole life, I don't like to have sex and I don't have sex with people, and I don't feel like messing with that kind of thing. So then, when I'm in withdrawal, I guess because I don't have sex with people, and I feel like really bad [sexual] hypersensitivity. It's also like it's extremely, extremely bad. And it's really embarrassing to me. It never goes away [when in withdrawal]. If I didn't get that, then I would stop. But because that happens, and nothing, nothing, nothing makes it better, like none of the medicines, none of the shit they give you, nothing makes it better. If I didn't have that problem then I would probably just stop and I'll be fine, but once that starts happening then I'm not fine anymore.”

-Kelly (white, female, 26 years old)

Similarly, when I asked another participant why he would use drugs in a highly visible location rather than trying to wait until a safer opportunity became available, he replied, “I have to, I can't get to the point where I'm shitting on myself.”

Thus, participants’ comments illustrate the ways that social capital and setting can moderate the need to engage in risk-involved activity as well as how judgements, and fears of deviating from social norms, are experienced by PWUIO as a force of constraint that effects their drug use choices.

## Discussion

This article examines the role of withdrawal in PWUIOs willingness to engage in risk-involved behavior. Findings show that withdrawal often functions as an important driver of a range of risk-involved behavior and that the proliferation of fentanyl has exacerbated the frequency and intensity of PWUIO’s withdrawal experiences likely driving greater exposure to risk. These results suggest that withdrawal, particularly in regions where fentanyl has become the dominant opioid available in illicit markets, should be taken more seriously both as a source of pain, dysphoria, and anxiety that PWUIO should not have to endure, and as a vector for engagement with risk-involved behavior.

However, findings did not align with the presentation of risk as a product of strictly individual-level irrationality as described in theories of addiction-as-disease. Rather, our data support Rhodes notion of contingent causation by demonstrating the important role of social and structural context on the kinds of risks that PWUIO encounter and their ability to address them. PWUIO’s choices were constrained both by structural forces that they had little to no control over, such as drug policy and the proliferation of fentanyl within the illegal drug supply, as well as by the economic and social contexts that their decisions about risk were made within. Participants with greater access to economic and social resources were better able to avoid or respond to withdrawal and consequently had less need to participate in risk-involved activity. In contrast, people who were homeless or unstably housed, experiencing economic difficulties, or who lacked a strong social network of people who could provide support generally had fewer options when addressing withdrawal and a greater need to take chances. Thus, PWUIO’s ability to avoid risk was moderated by the same kinds of socio-economic forces that moderate risk in other settings.

The risk environment that PWUIO lived and used drugs in was complex and dynamic, with different kinds of risks (policy, economic, social) overlapping and interacting with each other such that decisions could not easily be divided into “risk-involved” and “not-risk-involved” options. This aligns with the work of Giddens and others whose research shows the ways that structural and institutional forces (such as government, the economic system, and the criminal justice system) wield power over individuals, reducing their capacity for agency and autonomy [27, 58–66]. It also aligns with ethnographic work that shows how people who use illegal drugs, particularly highly criminalized and stigmatized drugs like heroin, must often make decisions from very limited, and often dangerous options [57, 67–71].

Claims that risk behavior by PWUIO is the result of their inherent irrationality are also reliant upon the false assumption that normative values are in fact universal [21, 22]. As scholars of risk, like Mary Douglas and Deborah Lupton have noted, different groups and individuals understand, experience, and assess risk and reward differently, and thus, they cannot be understood according to a singular metric [30, 72]. That PWUIO understood risks and benefits through their own subjective position as people who use drugs that are both highly criminalized and stigmatized was particularly clear in our data.

Our data also show that PWUIO engage in the same kinds of cost/benefit analyses when addressing withdrawal that most people use to make decisions, and while their choices may not align with the values of people who do not use drugs, they are logical and rational. Moreover, our data demonstrate the significant pain and anxiety produced by withdrawal and thus support the rationality of actions taken, even those that involve risk, to avoid or eliminate it. People do all kinds of things to escape pain, and when their options are sufficiently constrained, their actions are more likely to involve risk. Indeed, recent attempts by chronic pain patients to obtain illegal opioids after being cut off by their doctors [73–76] suggest there is nothing unique about “addicts” that accounts for their willingness to engage in more risk-involved activities when their access to less risky avenues is blocked or removed. In fact, it could be argued that these behaviors are actually health seeking by definition [77] and framing them as such may be a useful and productive means of challenging stigma. Moreover, discourses that position withdrawal as a minor discomfort, comparable to the flu, and easily ignored help to legitimize the notion that PWUIO are acting irrationally by engaging in risk-involved behavior to avoid or eliminate it.

Because of their focus on individual pathology to explain risk behavior, theories of addiction-as-disease work to medicalize and other PWUIO and thus add to the already-significant levels of stigma against them. They also devalue attempts at structural change such as ending the War on Drugs by obscuring the role of drug policy as a force of harm in the lives of people who use drugs and similarly weaken arguments for harm reduction initiatives, like drug testing and safe supply, that are based on the autonomy of people who use drugs and the notion that they will make healthier choices when given the tools to do so [78–81].

Since our data reflect the importance of context in the creation of risk, which we argue has been devalued in public health discussions of drug use risk, our recommendations are focused primarily on the risk environment rather than attempts at changing individual behavior. Specifically, our findings support the greater use of programs that reduce withdrawal among PWUIO such as low-threshold medication for opioid use disorder (MOUD) programs. Research shows that many PWUIO use MOUD as a strategy to reduce the risks of active drug use, in part by avoiding withdrawal, rather than to achieve abstinence [82, 83]. Yet, because most MOUD programs in the USA utilize punitive drug testing and require daily attendance for patients who continue using drugs, they are often avoided by such individuals, as reflected in MOUDs low rates of use and retention [84–87]. Indeed, many participants in this study reported difficulty remaining on MMT because of the strict regulatory environment. A low-threshold approach grounded in harm reduction rather than abstinence could significantly reduce the need for PWUIO to engage in the kinds of risk-involved behavior described here. Such programs have been utilized successfully in Canada and Spain and demonstrate better rates of retention and higher levels of patient satisfaction and quality of life [88–92]. MOUD programs in the USA should also develop and adopt induction and dosing protocols that address the higher tolerances associated with fentanyl use and ensure that withdrawal is adequately and quickly eliminated [93–95].

Low-barrier opioid distribution and safe supply programs that offer pre-tested drugs of a specific quantity should also be initiated in the USA and elsewhere to reduce peoples’ reliance on the unreliable and risk-involved illegal market. Maintenance programs using injectable diacetylmorphine rather than methadone demonstrate greater reductions in illegal opioid use and improved retention rates [96]. Similarly, “heroin compassion clubs” that allow eligible individuals to purchase pharmaceutical grade opioids [97] would provide PWUIO with a way out of cycle of withdrawal and risk described here.

Yet, since the risks PWUIO faced were nearly always linked to the context of prohibition/criminalization, it is drug policy reform that is most likely to reduce risks for PWUIO. As many have argued, and as our data support, it is the policy context of prohibition/criminalization that relegates PWUIO to a dangerous and unpredictable black market [98–101]. As such, the most effective and ethical means of reducing risk is to legalize and regulate drugs like heroin. If PWUIO were able to obtain them legally, safely, and at a price not artificially inflated by prohibition—as people who use drugs like alcohol and tobacco are currently able—withdrawal and the risks it engenders would likely be reduced dramatically.

This article has some important limitations. First, interviews were conducted in the NYC area which may have impacted the experiences of participants in our sample. The illicit opioid market in NYC has experienced a greater proliferation of fentanyl than many other places and that may have affected their views on withdrawal. Moreover, the specific context of US drug policy, policing, and access to services as well as the unique structure of NYC drug markets should also be noted in any analysis of our data. It is likely that the experiences of PWUIO in very urban areas like NYC differ from those in more rural settings. Similarly, the ways that drug use and people who use drugs are constructed are subject to cultural differences and may differ by location or by cultures within locations. For example, while dominant in the USA, the brain disease model is not as widely accepted in the UK where the individual drug user is often seen as a rational actor [81]. As such, this analysis should be seen within its US context [81]. Lastly, and as noted in the Methods section, this study utilized a situated methodology that informed the data collection and analysis. However, we do not believe that any of these issues significantly affected the themes that emerged in our data.

## Conclusions

In conclusion, we argue that withdrawal should be taken more seriously both from an ethical perspective, to lessen the suffering of fellow human beings, and also as an important catalyst of risk behavior. Moreover, we argue that judgements about the irrationality of PWUIO are reductive and abstracted from the complexity of their actual lives, and similarly, that the use of risk behavior as a marker of pathology and evidence supporting the ontological distinctness of “addicts” is problematic.

## Data Availability

The datasets used and/or analyzed during the current study are available from the corresponding author on reasonable request.
